# Supplementary material for: Nested retrotransposition in the East Asian mouse genome causes the classical nonagouti mutation
Source: Commun Biol. 2019 Aug 2;2:283. doi: 10.1038/s42003-019-0539-7 (PMC6677723; doi:10.1038/s42003-019-0539-7)
Supplement: Supplementary file 1 — Supplementary Information [file 42003_2019_539_MOESM1_ESM.pdf]

```
Unknown     TGTCAGAGACCATCCCGTGAGAAAGTGAGCCCTTCACAATCTCCCTAGCAGGCCAAATGG  60
Serpina3a   ..........A..A.T............TT.................A............  60
C6          .............-..............T...............T..A..A.T....G..  59
Ednrb^s     ..............................................G.............  60

Unknown     CCTTGTACTGAGAGCTGGTTGTCACCCCCCTTTCCTCCCTATTCCTTTCCTGGCACCTGA 120
Serpina3a   .........A..........A...........-................T.......... 119
C6          .....A...A...........C.......T.--.....T......C.....AA.....A. 117
Ednrb^s     ............................................................ 120

Unknown     GGCTGTAAAAGCTGAATTATAGTCCCCTCTTCCCTATCTCTTCCTGAGTTCCCATGCCAT 180
Serpina3a   ................................T.......................A... 179
C6          .A........TT.....A.C......................T.C...........A... 177
Ednrb^s     ...........---------------..............................A... 165

Unknown     CCAAGGACATGAGTTACGCCTGAGCCTAGCCTGACCCCCAAGGCTGTCAAGGAGGATCGA 240
Serpina3a   .............C.G..........C.......T......................... 239
C6          .............C.GT.........C...T....T...........TG.........A. 237
Ednrb^s     ..........................C................................. 225

Unknown     TGTTCCAGAGATAAGATCCAGAGTGCCCGCTGCCTGGCGCCTGACTTCGGCCCCCATGTC 300
Serpina3a   ........G................................................... 299
C6          G.C..TG..........G..A......T.....T..............A........... 297
Ednrb^s     ............................A............................... 285

Unknown     AGCAGATGCCCACTTCTTTGTTCTTTGTATAATTCTCCCTCGACCCCTCCCATATTCCCC 360
Serpina3a   ............................................................ 359
C6          .....TC.T..TT.C.C............C..C..................C....TT.. 357
Ednrb^s     ........................................A................... 345
                                      TATA box            U3|R
Unknown     GCGATGTATGCTTTAAAAAGAAGGCACCTCAGCCTAATAAACGAGACCTTGATAGGTTCA 420
Serpina3a   ...............T.................G...............C....A..--. 417
C6          ...............T..G...A..........T.......T................T. 417
Ednrb^s     ............................................................ 405
                 R|U5                                 poly(A)signal
Unknown     ATCTACTTGGTCCTCCGACTCTTCTTTCTTTTACTCCCATTTCCTTCCAGGTTTGCGGTC 480
Serpina3a   CC..T.C.....-...T.T.TC...............................C...... 476
C6          .................G..-C..........C....................C...... 476
Ednrb^s     ............................................................ 465

Unknown     CCCCTCGCACCCACGAATAACTGAGTCCCGCGGGACGGATA 522
Serpina3a   ..........G.............A...T............ 518
C6          ........................A................ 518
Ednrb^s     ........................A................ 507
```

Supplementary Figure 1 | Comparison between the sequence of the direct terminal repeat of the unknown element in the *a* allele and homologous sequences in the *Serpina3a* and *C6* mRNAs and the inserted sequence in the *s* allele. The positions of the candidate TATA box and poly (A) signal are indicated by open boxes with the corresponding names. The U3, R, and U5 regions are shown shaded in gray, and the borders are indicated by lines. The position of each sequence is shown on the right.

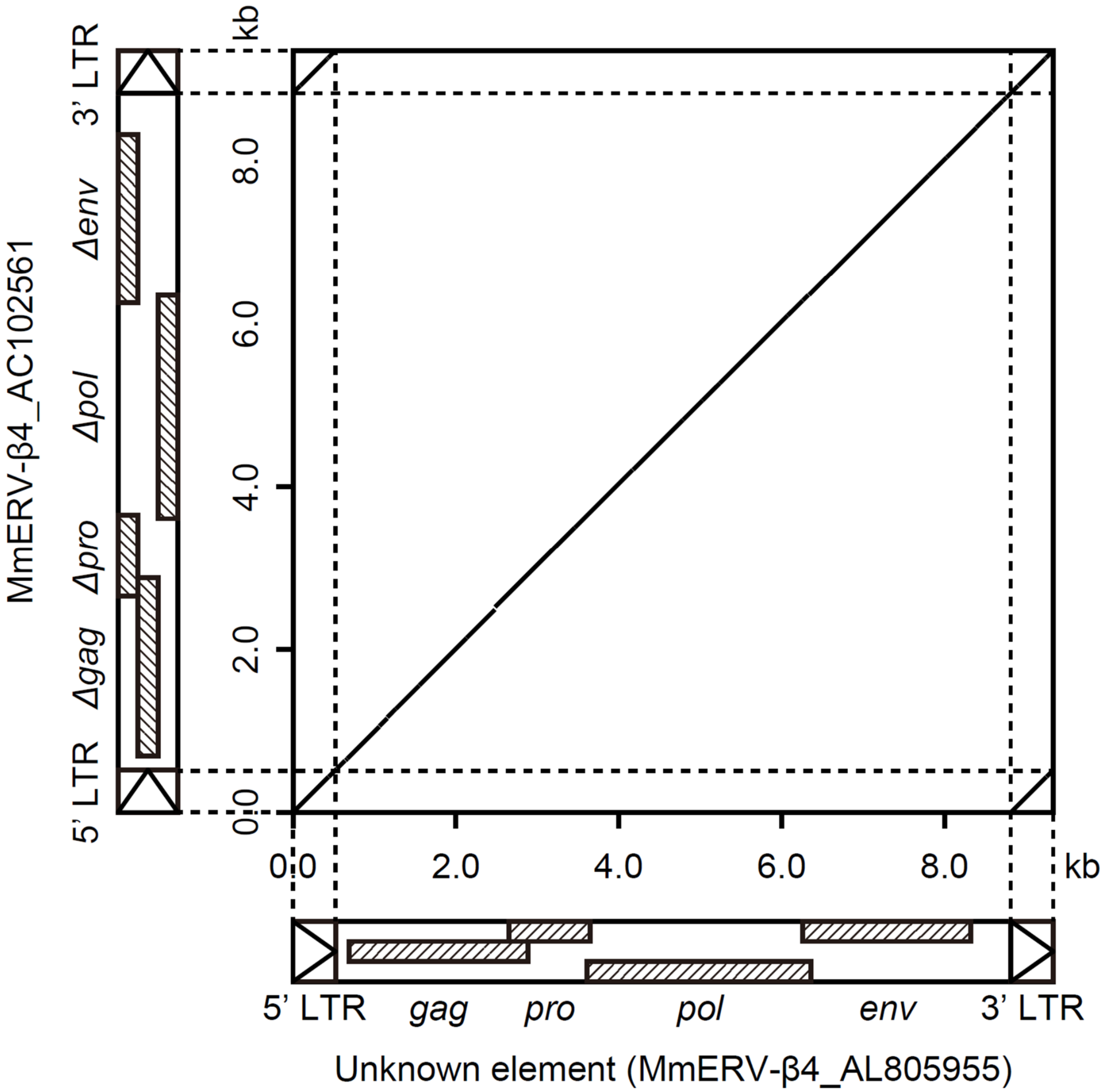

Supplementary Figure 2 | Dot plot of the unknown sequence in the *a* allele against the reference β4 sequence. Comparison of the unknown sequence in the *a* allele (horizontal axis) and the reference β4 (MmERV-β4_AC102561) sequence (vertical axis). The description is according to Fig. 2. LTR, long terminal repeat. kb, kilobases.

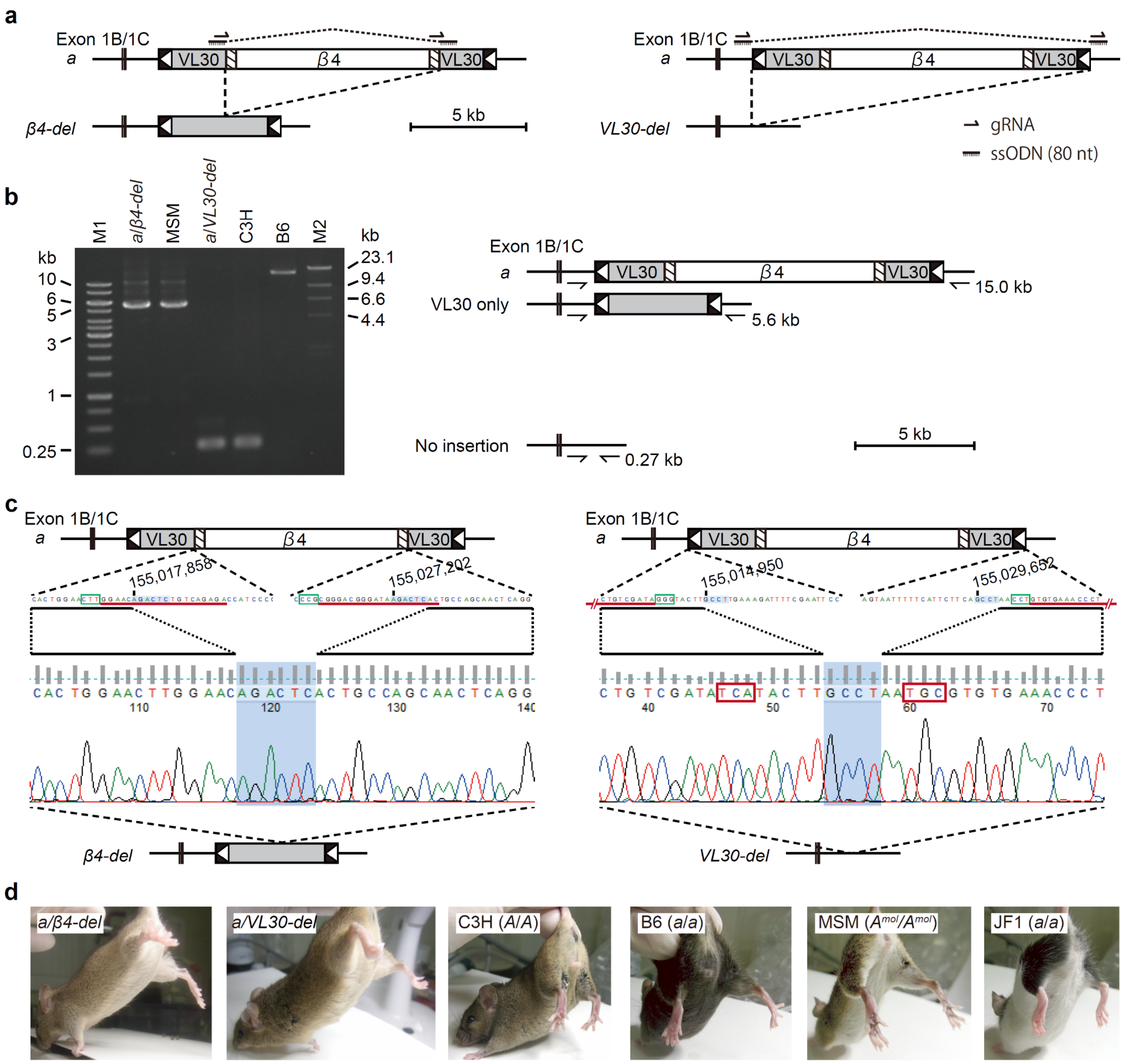

Supplementary Figure 3 | Generation of β4-del and VL30-del mice. a, Targeting scheme to delete the β4 (left) and the VL30 (right) insertions in the *a* allele. Scale bar = 5 kb. **b,** Gel-electrophoresis of the PCR-amplified genomic DNA fragments spanning the targeted site. A schematic illustration of the structure of each amplified DNA fragment is shown on the right. Primers used for genomic PCR are marked by arrows. Scale bar = 5 kb. **c,** Representative electropherograms from Sanger sequencing of the region spanning the targeted β4 (left) and VL30 (right) insertions. The directly duplicated sequence on both sides of the deleted endogenous retroviral sequence is highlighted by a blue background. The protospacer adjacent motif (PAM) (green box) and the gRNA sequences (red lines) are shown above the electropherogram. In the targeting of the VL30 insertion, the PAM sequence was modified from the original genomic sequence to an unrelated sequence (red boxed) to avoid re-cutting by the Cas9 nuclease of the target gRNA. **d,** Ventral views of adult β4-del (*a*/*β4-del*) and VL30-del (*a*/*VL30-del*) heterozygous mutant mice and that of B6 (*a*/*a*), MSM (A^{mol}/A^{mol}), and JF1 (*a*/*a*) mice. Both targeted deletions resulted in a cream-colored ventrum, whereas the MSM mouse has a much paler ventrum. kb, kilobases.

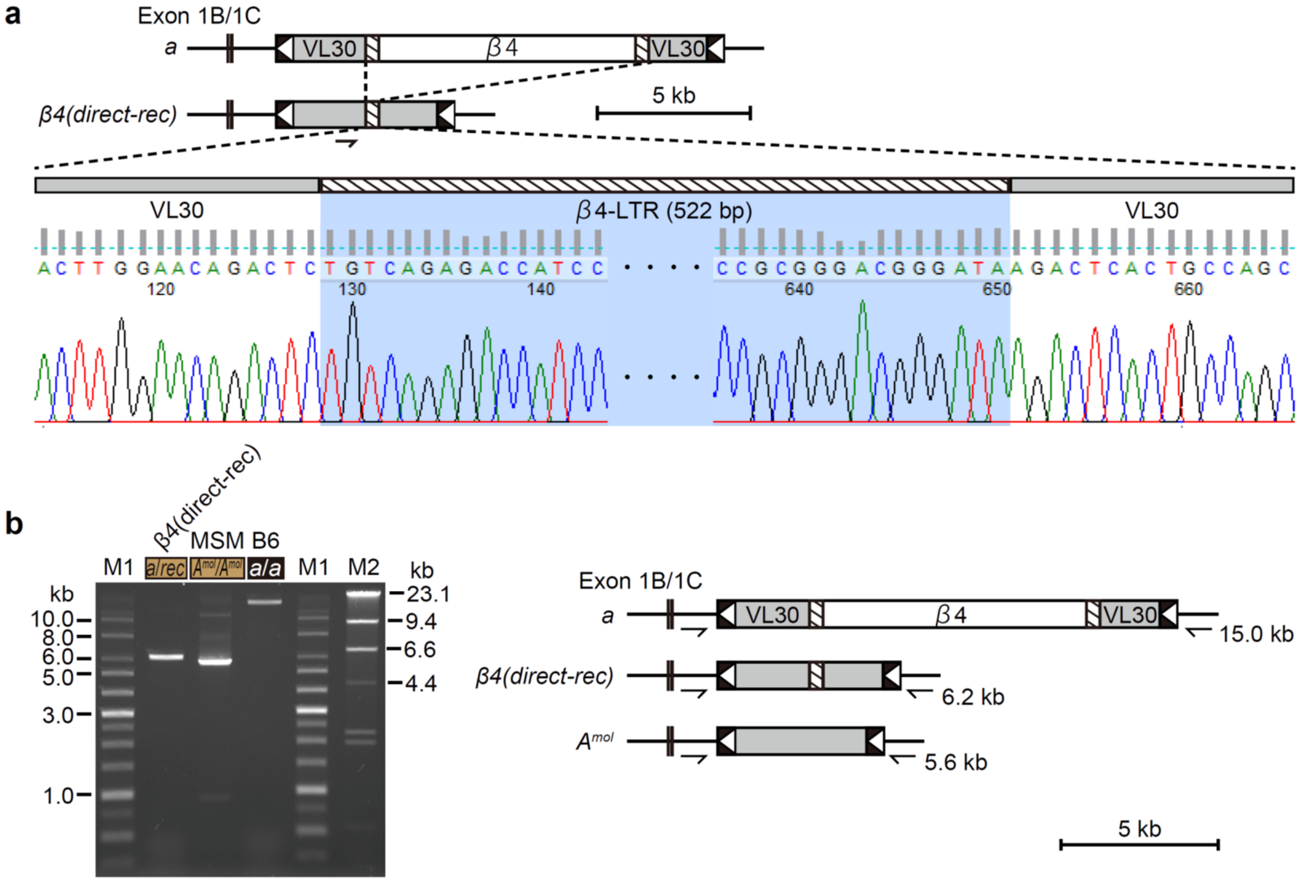

Supplementary Figure 4 | Generation of β4(direct-rec) mice. a, Top; Schematic illustration of the structure of the a^t allele. Primer used for Sanger sequencing is shown with an arrow. Scale bar = 5 kb. Bottom; Representative electropherogram from Sanger sequencing of the solo β4 LTR insertion in the VL30 sequence in the a^t allele. The β4 LTR sequence is highlighted with a blue background. The central region of the β4 LTR is omitted. **b,** Gel electrophoresis of the PCR-amplified genomic DNA fragments spanning the insertions in the *agouti* locus. The *β4(direct-rec)* allele shows a longer PCR product than the MSM allele, which contains a solo VL30 insertion. Schematic illustrations of the structures of each amplified DNA fragment are shown on the right. Primers used for genomic PCR are shown with arrows. Scale bar = 5 kb.

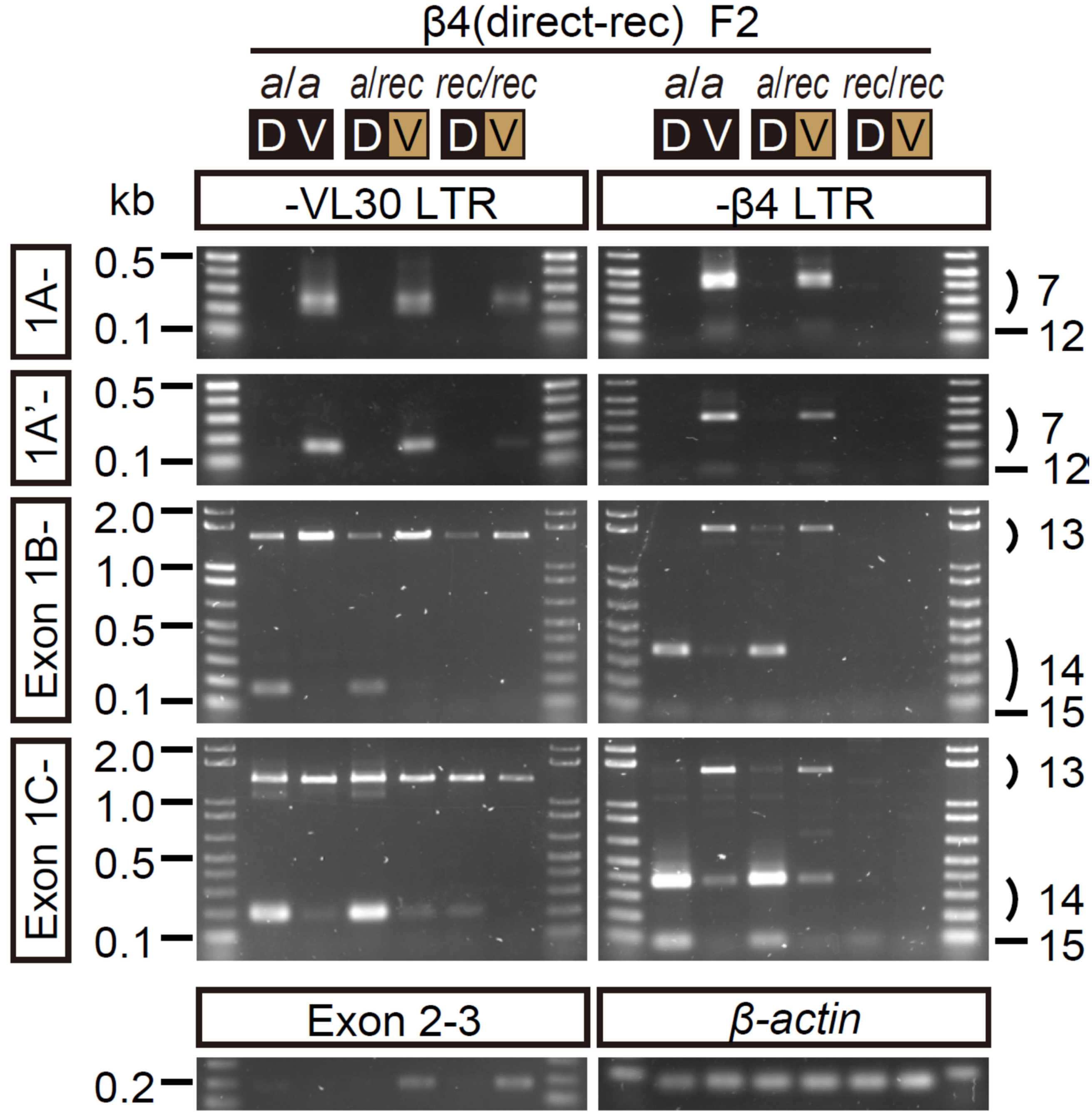

Supplementary Figure 5 | RT-PCR of the *agouti* transcripts in the dorsal and ventral skins of neonatal β4(direct-rec) mice. F2 littermates [*a*/*a*, *a*/*β4(direct-rec)* and *β4(direct-rec)*/*β4(direct-rec)*] were used for the analyses. The *agouti* transcripts were amplified from exons 1A, 1A′, 1B, and 1C to the VL30 LTR or the β4 LTR. Transcript numbers according to those in Fig. 4. are shown on the right. The *agouti* transcripts from exon 2 to 3 were also amplified. β-actin was used as an internal control. D, dorsal skin; V, ventral skin.

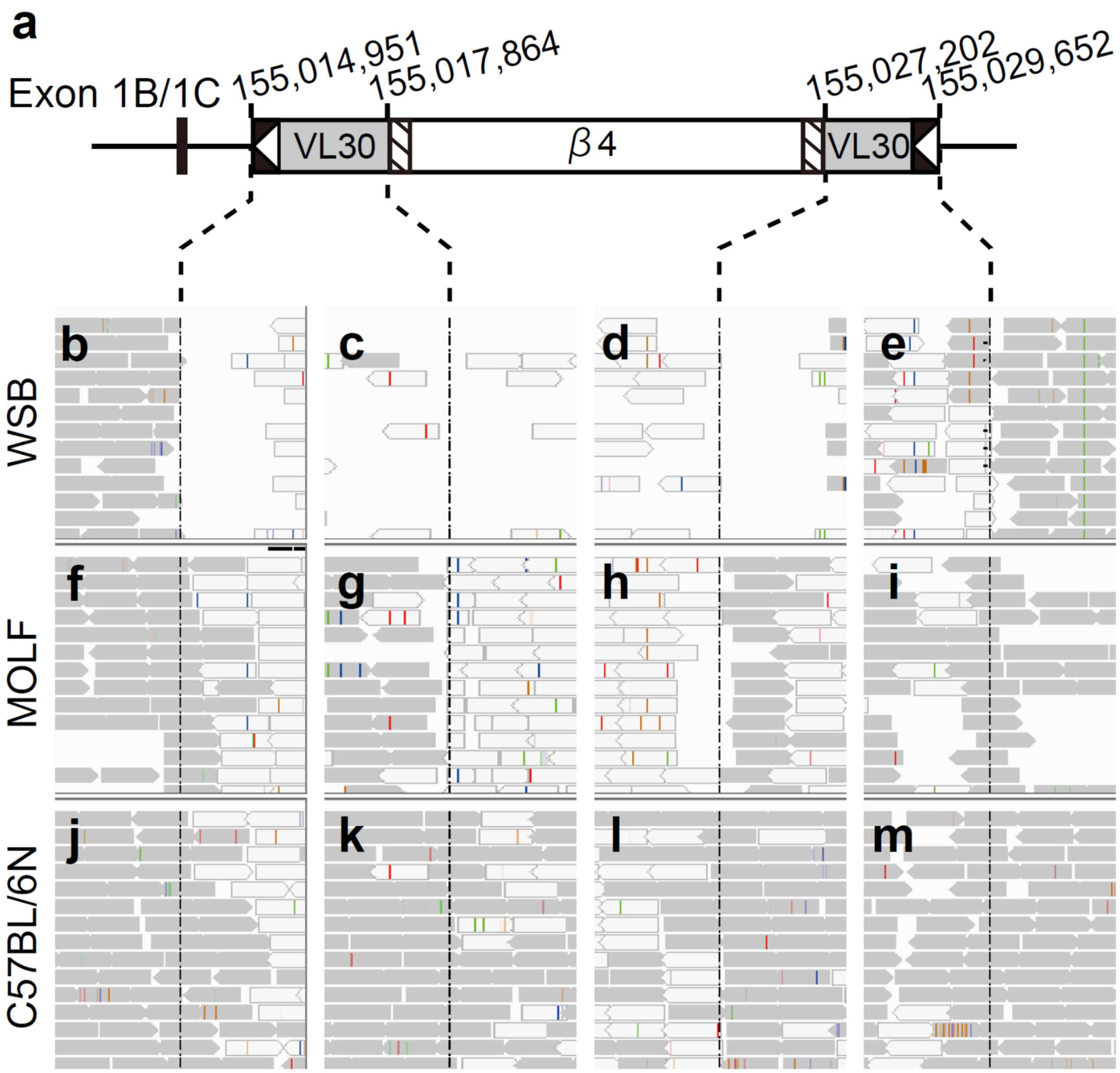

Supplementary Figure 6 | Representative mapping of NGS reads to sites of the β4 and the VL30 insertions in the *a* allele. a, Schematic representation of structure of the VL30 and the β4 elements in the *a* allele. **b–m,** Mapping of the NGS data[1] from WSB (**b–e**), MOLF (**f–i**). and C57BL/6N (**j–m**) strains spanning the sites of the VL30 (left sides: **b, f, j**; right sides: **e, i, m**) and β4 insertions (left sides: **c, g, k**; right sides: **d, h, l**) in the *a* allele. kb, kilobases.

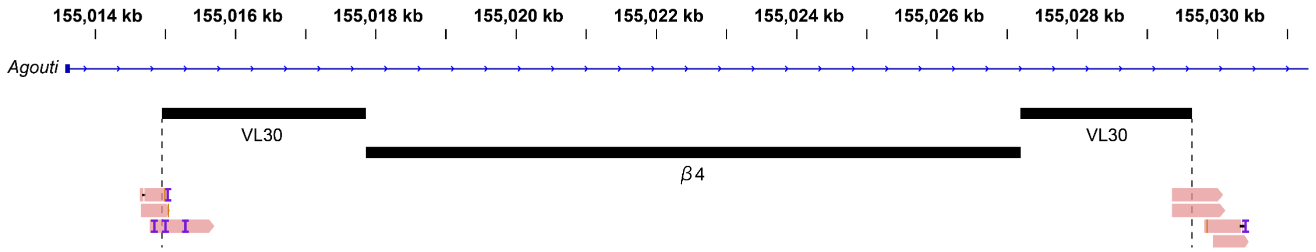

Supplementary Figure 7 | Mapping of capillary sequencing data around the insertion of VL30 in the *agouti* locus in the MSM strain. Capillary sequencing data[2] derived from MSM genomic DNA was mapped onto the B6 genome. Mapped sequences are indicated as pink bars. The regions of the VL30 and β4 elements are indicated by black bars.

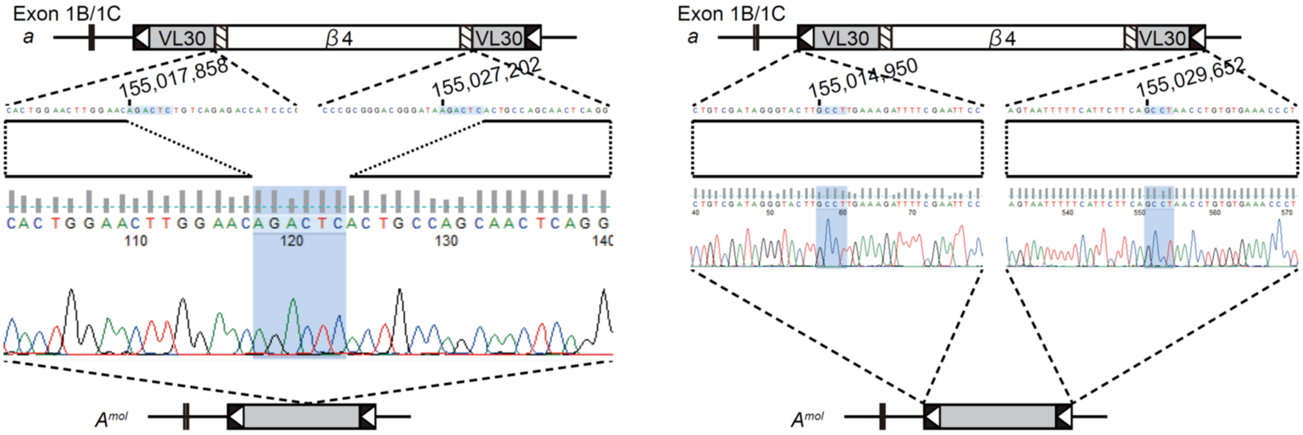

Supplementary Figure 8 | Sequencing of the VL30 in the *agouti* loci of wild-derived strains. Representative electropherograms from Sanger sequencing of the region spanning the sites of the β4 (left) and VL30 (right) insertions in the MSM (A^{mol}/A^{mol}) strain.

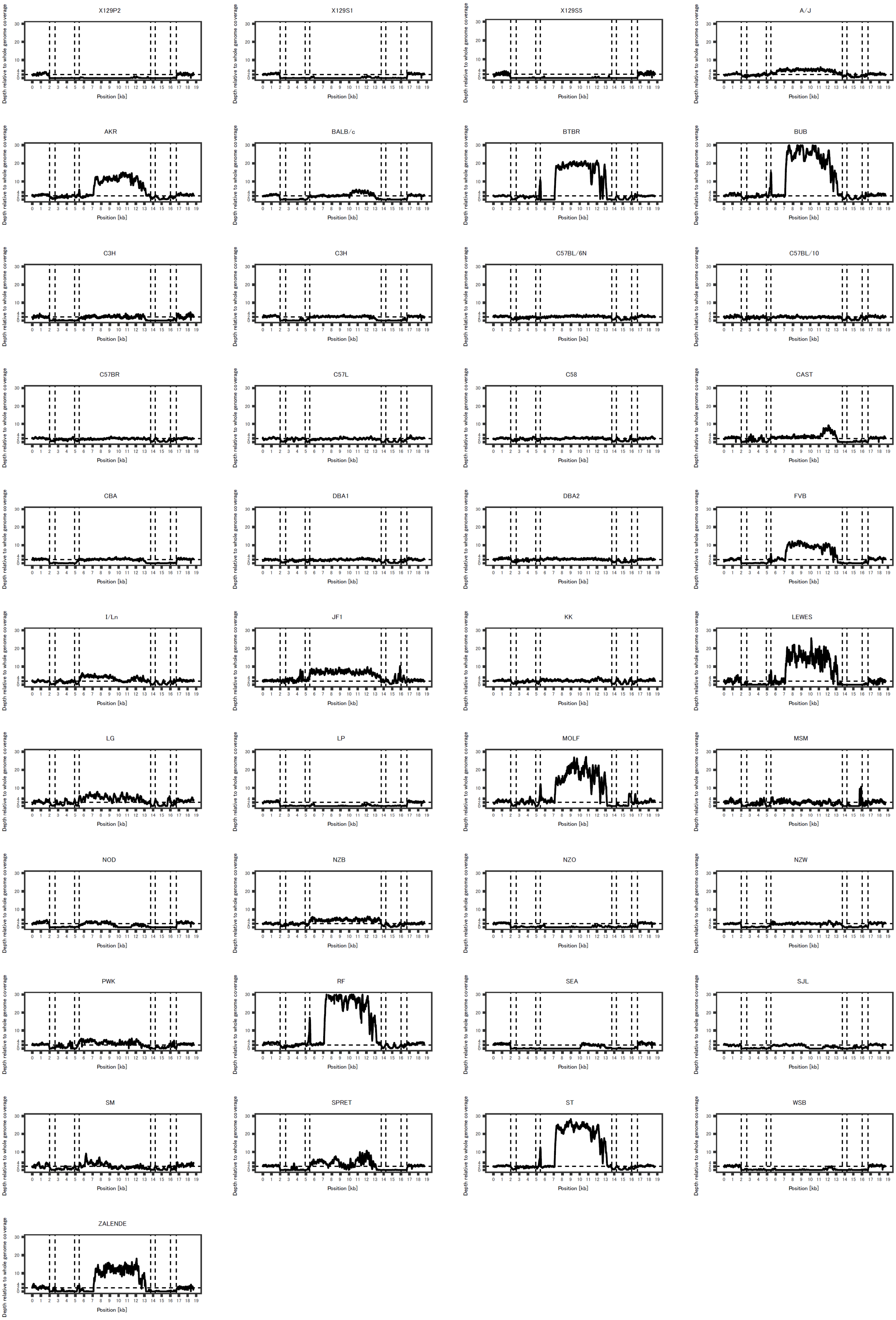
X129P2
X129S1
X129S5
A/J
AKR
BALB/c
BTBR
BUB
C3H
C3H
C57BL/6N
C57BL/10
C57BR
C57L
C58
CAST
CBA
DBA1
DBA2
FVB
I/Ln
JF1
KK
LEWES
LG
LP
MOLF
MSM
NOD
NZB
NZO
NZW
PWK
RF
SEA
SJL
SM
SPRET
ST
WSB
ZALENDE
Depth relative to whole genome coverage
Position [kb]

Supplementary Figure 9 | Depth of NGS reads across the β4 and the VL30 insertions in *agouti* loci in various inbred strains. For each strain, we determined the sequencing depth across the β4 and the VL30 insertions and adjacent 2 kb of flanking sequences (chr2:155,012,951–155,031,651) relative to the coverage of the whole genome. The relative values were multiplied by 2 to determine copy number. The horizontal dashed line indicates a depth of 2. The vertical dashed lines indicate the positions of both sides of the 5' and 3' LTRs of the β4 and the VL30. The depths of the LTR regions are underestimated because of low mapping quality. kb, kilobases.

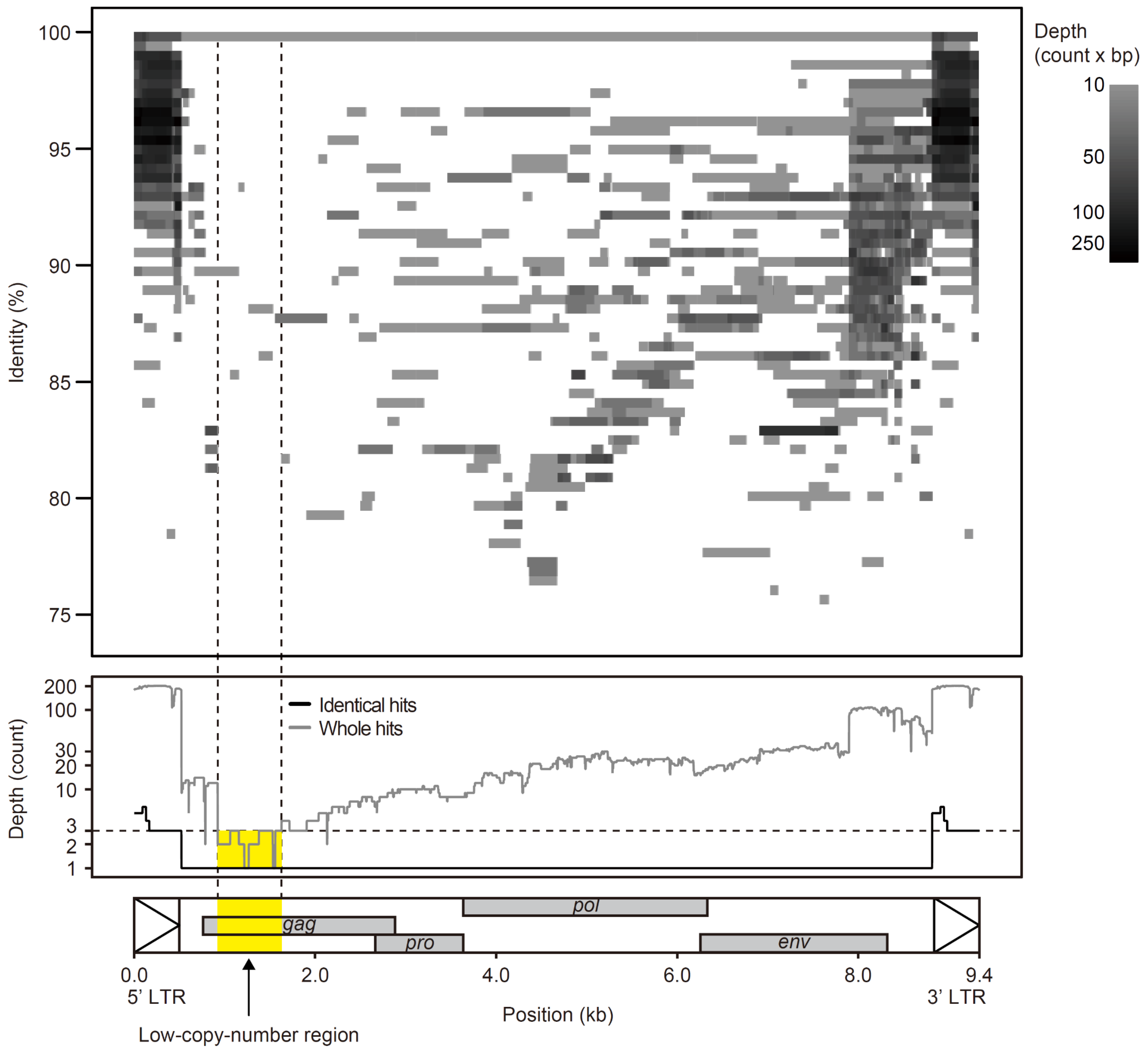

Supplementary Figure 10 | Recruitment plot of homologous sequences of β4 in the *a* allele. BLAST hits with >70 % identity aligned against the β4 in the *a* allele. The *x*-axis shows the hit position along the sequence, and the *y*-axis indicates the percent identity of the hit, ranging from 75 to 100 % (upper panel), and the depth of each hit (lower panel). Whole hits (gray line) and hits with 100 % sequence identity (black line) are indicated. Schematic representation of structure of β4 in the *a* allele is indicated on the bottom with its low copy-number region (yellow). LTR, long terminal repeat. kb, kilobases. bp, base pair.

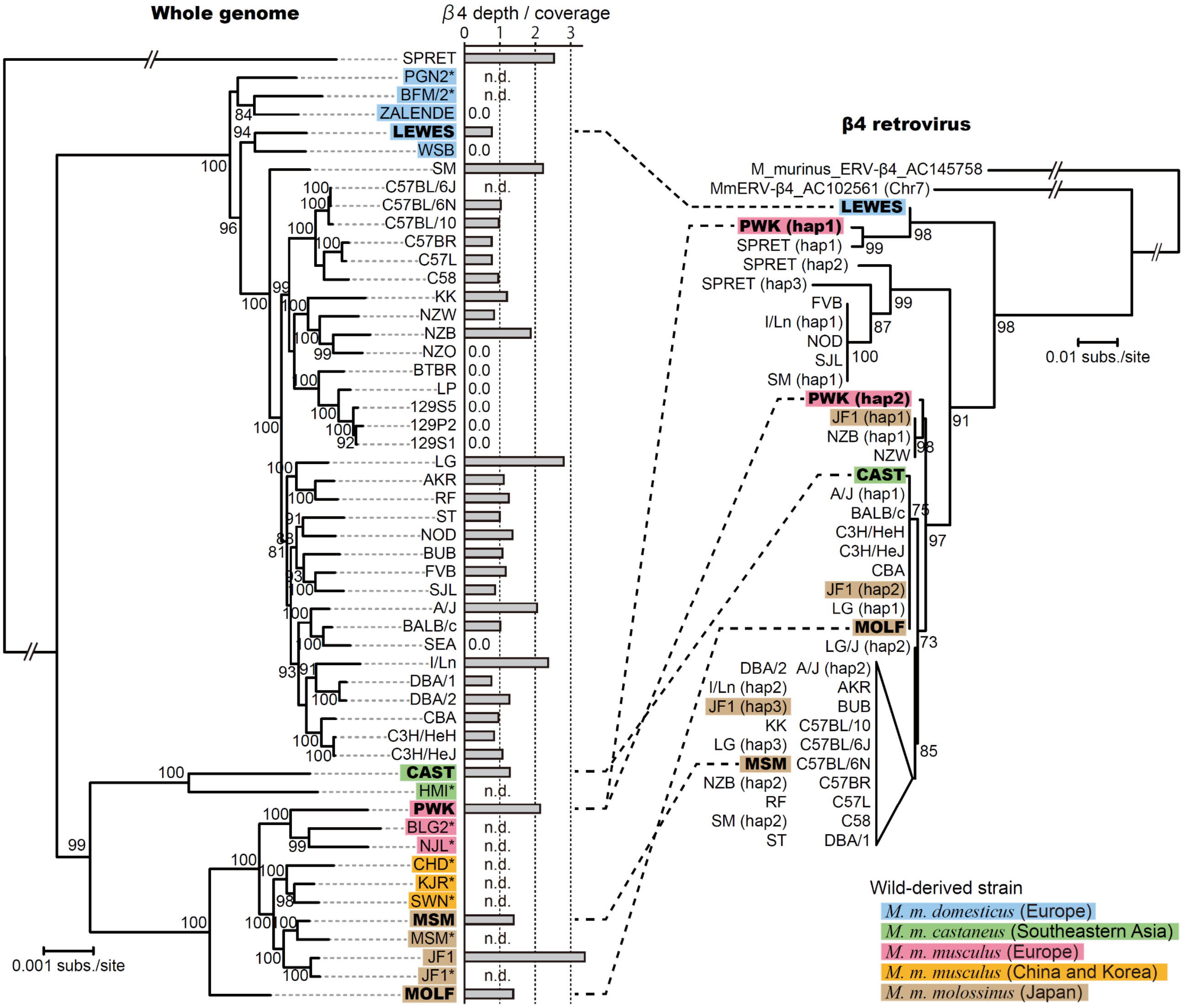

Supplementary Figure 11 | Tanglegram of the host and β4 retrovirus. Maximum likelihood neighbor-joining trees based on alignments of the sequence of all autosomal chromosomes (left) and the haplotypes of the β4 sequence (right). Wild-derived strains (bold letters) are connected with dashed lines between the phylogenetic trees. Left: The *M. spretus*-derived SPRET/EiJ (SPRET) was used as an outgroup. Right: a gray mouse lemur (*Microcebus murinus*) endogenous retrovirus (M_murinus_ERV-β4_AC145758) and the reference β4 retrovirus (MmERV-β4_AC102561) were used as an outgroup. Middle: the copy numbers of β4 are shown as the average depth in the low copy-number region of β4 divided by the whole-genome coverage. The description is according to Fig. 5a. Scale bar = 0.001 (left) and 0.01 (right) nucleotide substitutions per site. n.d., no data available.

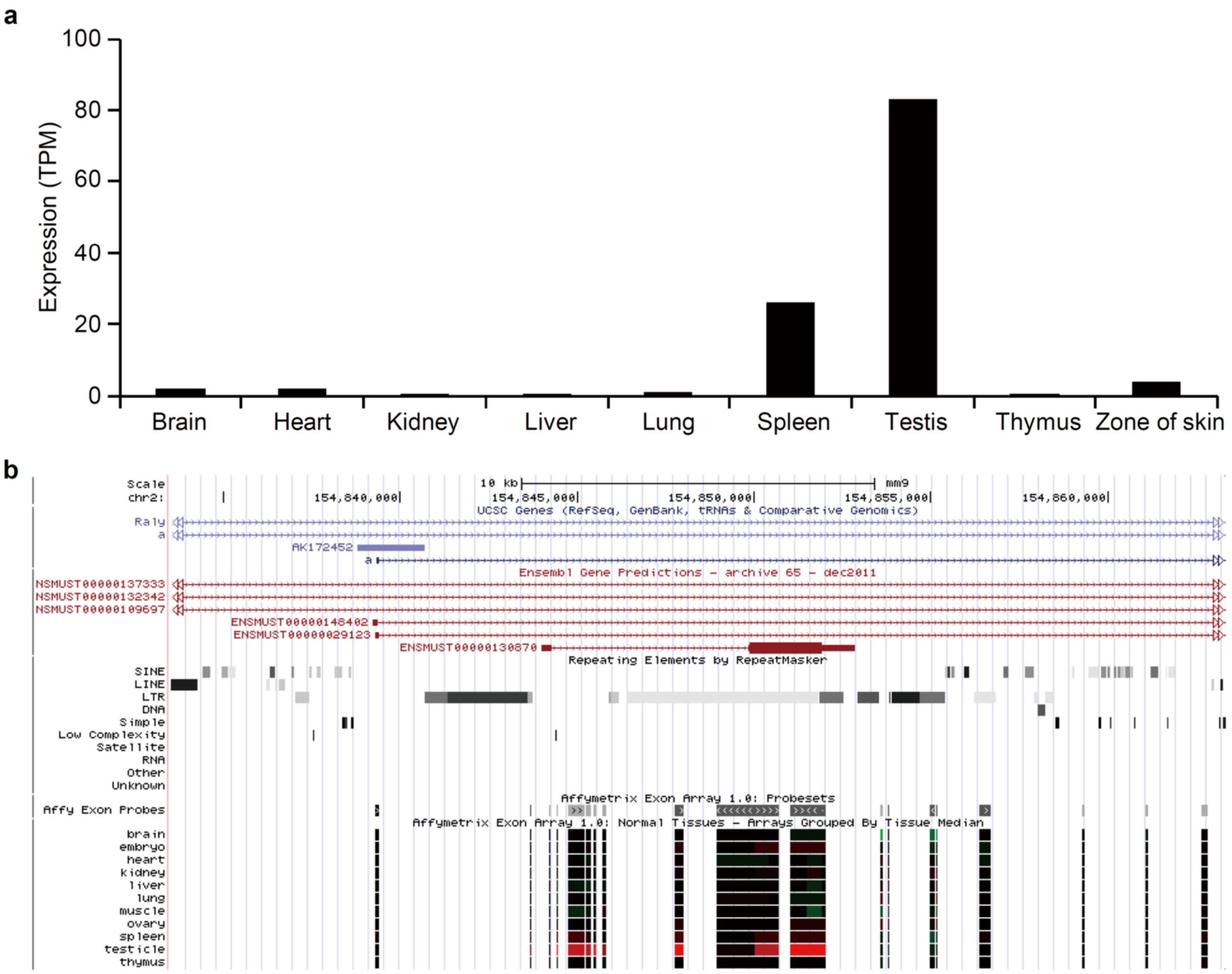

Supplementary Figure 12 | Tissue-specific expression of β4 endogenous retrovirus in the *a* allele. a, RNA-seq data (GSE74747)[3] of the *Gm14226* gene (ENSMUSG00000084897 - Ensembl 91) in nine tissues from an adult male B6 mouse. Data were obtained from Expression Atlas. **b,** Microarray data[4] of the *Gm14226* gene (ENSMUST00000130870 - Ensembl 65) in 11 tissues from Affymetrix Mouse Exon 1.0 ST arrays. Screenshot of UCSC Genome Browser was captured using NCBI37/mm9 assembly.

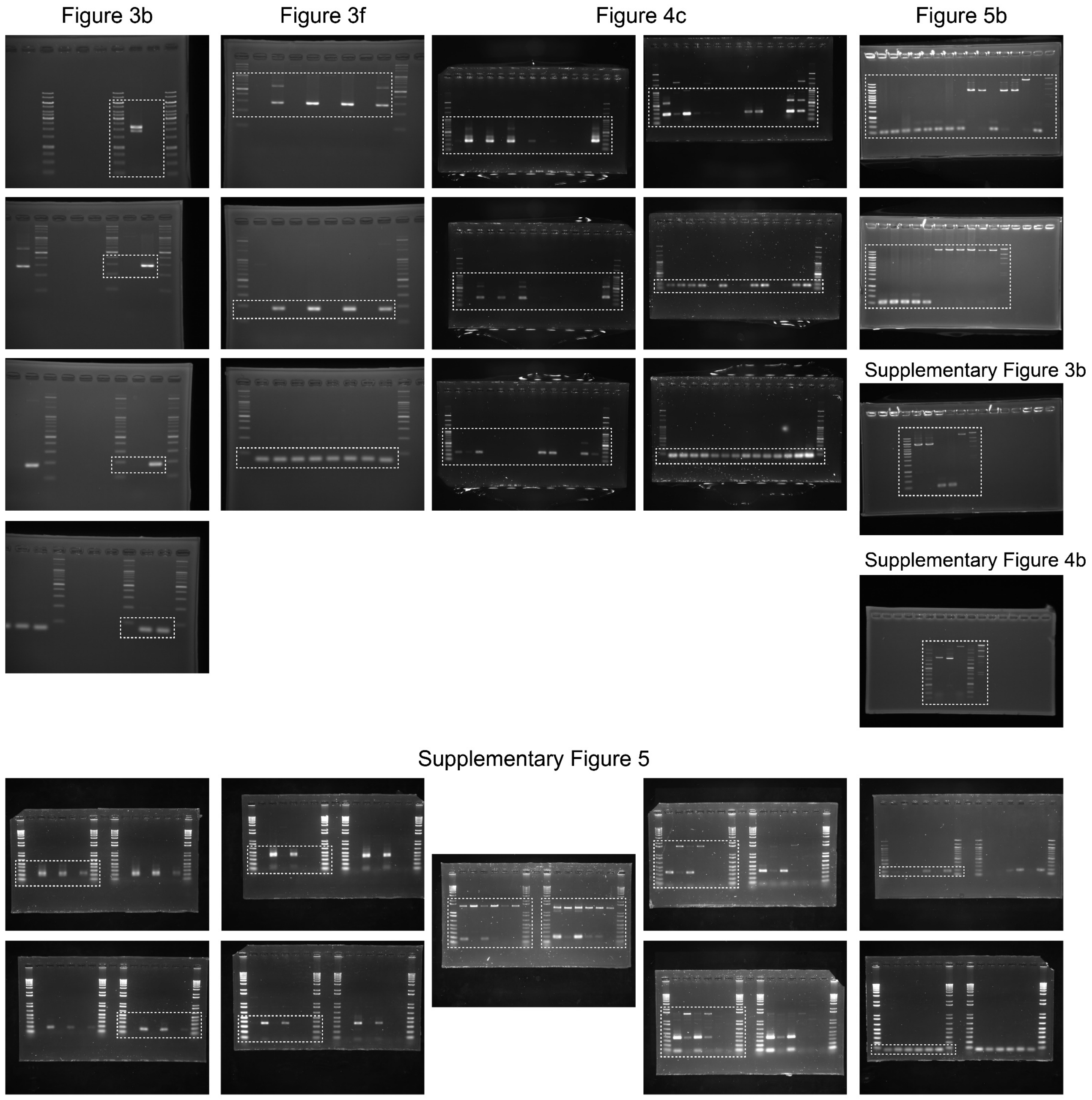

Supplementary Figure 13 | Original uncropped images of agarose gels. White broken boxes indicate the borders of the cropped images presented in the manuscript.

Supplementary Table 1 | Summary of primer and oligonucleotide sequences.

Name	Sequence	Length [nt]	Chr.	Strand	Start	End	Experiment
Agouti-exon1A F2	TCAGAAGAGGGAGTCATCAGC	21	2	+	154,951,250	154,951,270	RT-PCR
Agouti-exon1Ad F1	CGATTTCTGATGAATCCTGGAAC	23	2	+	154,952,550	154,952,572	RT-PCR
Agouti-exon1B F1	CTGGGACGAGTCTGAACCTAC	21	2	+	155,013,452	155,013,472	RT-PCR
Agouti-exon1C F1	AGACATTCTGGCCTGGCTTC	20	2	+	155,013,584	155,013,603	RT-PCR
Agouti-exon2 F1	GGCTTCCTGTGCTTCTTCAC	20	2	+	155,045,658	155,045,677	RT-PCR
Agouti-exon3 R2	GAAGACCTCTTCCGCTTCTC	20	2	-	155,047,717	155,047,736	RT-PCR
VL30 LTR R1	GGAACAGCCTCTCTCAATTCC	21	2	-	155,014,903	155,014,923	RT-PCR
MmERV-B4 LTR 6R	GTTATTCGTGGGTGCGAG	18	2	-	155,018,347	155,018,364	RT-PCR
ERVB4-1_env 1R	TCTTCTTTGAGGGCTGCACATA	22	2	-	155,025,746	155,025,767	RT-PCR
βactin F	TGACAGGATGCAGAAGGAGA	20	5	-	142,904,102	142,904,121	RT-PCR
βactin R	CGCTCAGGAGGAGCAATG	18	5	+	142,903,922	142,903,939	RT-PCR
T7-ERVb4R-opti-gRNA 1F	GGATCCTAATACGACTCACTATAGGGGTGAGTCTTATCCCGTCCCGGTTTAAGAGCTATGCTGG	64	N/A	N/A	N/A	N/A	CRISPR
T7-ERVb4L-opti-gRNA 1F	GGATCCTAATACGACTCACTATAGGGTCTCTGACAGAGTCTGTTCCGTTTAAGAGCTATGCTGG	64	N/A	N/A	N/A	N/A	CRISPR
T7-VL30-opti-gRNA1	GGATCCTAATACGACTCACTATAGGGGTTCCACAGCTCTGTCGATAGTTTAAGAGCTATGCTGG	64	N/A	N/A	N/A	N/A	CRISPR
T7-VL30-opti-gRNA2	GGATCCTAATACGACTCACTATAGGGTCAAACCCAGGGTTTCACACGTTTAAGAGCTATGCTGG	64	N/A	N/A	N/A	N/A	CRISPR
T7-gRNA-PCR-R	AAAAAAGCACCGACTCGGTGCC	22	N/A	N/A	N/A	N/A	CRISPR
Null-ERVb4-37bp-AGACTC-37bp	CAAGTGGGCGGATGCCTCCCTCACTGGAACTTGGAACAGACTCACTGCCAGCAACTCAGGGTGG TGCCACCTTCTGAGAA	80	2	+	155,017,821	155,027,244	CRISPR
VL30-del oligo	GAGAGAGGCTGTTCCACAGCTCTGTCGATATCATACTTGCCTAATGCGTGTGAAACCCTGGGTTT GATTCCCCAGCATCA	80	2	+	155,014,909	155,029,693	CRISPR
Agouti-VL30 5F	CCAAGTGAAAGGAATTGAGAGGCTGTTCCACAGCTCTG	38	2	+	155,014,893	155,014,932	Genomic PCR, Sequencing
Agouti-VL30 4R	TTGGGAATCACAAGCATACACCTCATATCCGGTTGG	36	2	-	155,029,833	155,029,868	Genomic PCR, Sequencing
MMVL30-ins.check F1	CTTTCTGTTACCTTGGTCAGATTGTTG	27	2	+	155,017,707	155,017,733	Sequencing
MMVL30-ins.check R1	TTTCACTTTAAGATAAGTGCCGGTTG	26	2	-	155,027,301	155,027,326	Sequencing

Supplementary Table 2 | Summary of inbred strains.

Strain name	Species	Subspecies	Type of lab strain	Strain origin (country)	Strain origin (province/state)	Strain origin (city)	Source Name (institution)	NGS data (institution)
129P2/OlaHsd	*Mus musculus*	na	Classical	Castle	na	na	JAX	Sanger
129S1/SvlmJ	*Mus musculus*	na	Classical	Castle	na	na	JAX	Sanger
129S5/SvEvBrd	*Mus musculus*	na	Classical	Castle	na	na	JAX	Sanger
A/J	*Mus musculus*	na	Classical	Castle	na	na	JAX	Sanger
AKR/J	*Mus musculus*	na	Classical	Castle	na	na	JAX	Sanger
Balb/cJ	*Mus musculus*	na	Classical	Castle	na	na	JAX	Sanger
BFM/2Ms	*Mus musculus*	*domesticus*	Wild-derived	France	Montpellier	na	NIG	NIG
BLG2/Ms	*Mus musculus*	*musculus*	Wild-derived	Bulgaria	Toshevo	na	NIG	NIG
BTBR T^{+} $Itpr3^{tf}$/J	*Mus musculus*	na	Classical	Unknown	na	na	JAX	Sanger
BUB/BnJ	*Mus musculus*	na	Classical	Other inbred strains	na	na	JAX	Sanger
C3H/HeH	*Mus musculus*	na	Classical	Castle	na	na	JAX	Sanger
C3H/HeJ	*Mus musculus*	na	Classical	Castle	na	na	JAX	Sanger
C57BL/10J	*Mus musculus*	na	Classical	C57-related	na	na	JAX	Sanger
C57BL/6NJ	*Mus musculus*	na	Classical	C57-related	na	na	JAX	Sanger
C57BR/cdJ	*Mus musculus*	na	Classical	C57-related	na	na	JAX	Sanger
C57L/J	*Mus musculus*	na	Classical	C57-related	na	na	JAX	Sanger
C58/J	*Mus musculus*	na	Classical	C57-related	na	na	JAX	Sanger
CAST/EiJ	*Mus musculus*	*castaneus*	Wild-derived	Thailand	na	Thonburi	JAX	Sanger
CBA/J	*Mus musculus*	na	Classical	Castle	na	na	JAX	Sanger
CHD/Ms	*Mus musculus*	*musculus*	Wild-derived	China	Chengdu	na	NIG	NIG
DBA/1J	*Mus musculus*	na	Classical	Castle	na	na	JAX	Sanger
DBA/2J	*Mus musculus*	na	Classical	Castle	na	na	JAX	Sanger
FVB/NJ	*Mus musculus*	na	Classical	Swiss	na	na	JAX	Sanger
HMI/Ms	*Mus musculus*	*castaneus*	Wild-derived	Taiwan	Hemei	na	NIG	NIG
I/LnJ	*Mus musculus*	na	Classical	Castle	na	na	JAX	Sanger
JF1/Ms	*Mus musculus*	*molossinus*	Fancy mice	Japan	na	Denmark Market	NIG	Sanger
KJR/Ms	*Mus musculus*	*musculus*	Wild-derived	Korea	Kojuri	na	NIG	NIG
KK/HIJ	*Mus musculus*	na	Classical	China & Japan	na	na	JAX	Sanger
LEWES/EiJ	*Mus musculus*	*domesticus*	Wild-derived	US	Delaware	na	JAX	Sanger
LG/J	*Mus musculus*	na	Classical	Other inbred strains	na	na	JAX	Sanger
LP/J	*Mus musculus*	na	Classical	Castle	na	na	JAX	Sanger
MOLF/EiJ	*Mus musculus*	*molossinus*	Wild-derived	Japan	Kyushu	Fukuoka	JAX	Sanger
MSM/Ms	*Mus musculus*	*molossinus*	Wild-derived	Japan	Shizuoka	Mishima	NIG	NIG
NJL/Ms	*Mus musculus*	*musculus*	Wild-derived	Denmark	Northern Jutland	na	NIG	NIG
NOD/LtJ	*Mus musculus*	na	Classical	Swiss	na	na	JAX	Sanger
NZB/BINJ	*Mus musculus*	na	Classical	Castle	na	na	JAX	Sanger
NZO/HILtJ	*Mus musculus*	na	Classical	Castle	na	na	JAX	Sanger
NZW/LacJ	*Mus musculus*	na	Classical	Castle	na	na	JAX	Sanger
P/J	*Mus musculus*	na	Classical	Castle	na	na	JAX	Sanger
PGN2/Ms	*Mus musculus*	*domesticus*	Wild-derived	Canada	Ontario	Windsor	NIG	NIG
PWK/PhJ	*Mus musculus*	*musculus*	Wild-derived	Czech Rep	Bohemia	Lhotka	JAX	Sanger
RF/J	*Mus musculus*	na	Classical	Castle	na	na	JAX	Sanger
SEA/GnJ	*Mus musculus*	na	Classical	Castle	na	na	JAX	Sanger
SEG/Pas	*Mus spretus*	na	Wild-derived	Spain	Granada	na	PAS	na
SJL/J	*Mus musculus*	na	Classical	Swiss	na	na	JAX	Sanger
SM/J	*Mus musculus*	na	Classical	Castle	na	na	JAX	Sanger
SPRET/EiJ	*Mus spretus*	na	Wild-derived	Spain	Cadiz Province	Puerto Real	JAX	Sanger
ST/bJ	*Mus musculus*	na	Classical	Other inbred strains	na	na	JAX	Sanger
SWN/Ms	*Mus musculus*	*musculus*	Wild-derived	Korea	Suweon	na	NIG	NIG
WSB/EiJ	*Mus musculus*	*domesticus*	Wild-derived	US	Maryland	Centerville	JAX	Sanger
ZALENDE/EiJ	*Mus musculus*	*domesticus*	Wild-derived	Switzerland	na	Zalende	JAX	Sanger

Supplementary Table 3 | Genome coverage and depth and estimated copy numbers of the β4 sequence among inbred strains.

Sample	Total_Depth	LCN_Depth	LCN_Depth (hard filtered)	Internal_ Depth	LCN_Depth/Total_Depth	LCN_Depth(hard filtered)/Total_Depth
129P2/OlaHsd	37.49	0.10	0.05	1.37	0.00	0.00
129S1/SvImJ	56.61	0.18	0.18	4.20	0.00	0.00
129S5/SvEvBrd	17.14	0.00	0.00	0.48	0.00	0.00
A/J	48.27	99.94	98.95	99.21	2.07	2.04
AKR/J	50.00	55.40	55.09	213.91	1.10	1.10
BALB/cJ	53.93	55.52	54.82	69.50	1.02	1.01
BTBR T^{+} $Itpr3^{tf}$/J	78.12	0.00	0.00	491.64	0.00	0.00
BUB/BnJ	42.03	45.63	45.04	372.43	1.08	1.07
C3H/HeH	13.47	14.24	14.21	13.00	1.05	1.05
C3H/HeJ	55.43	60.56	59.56	55.68	1.09	1.07
C57BL/10J	25.50	27.28	26.97	24.41	1.06	1.05
C57BL/6NJ	52.20	54.09	53.25	59.05	1.03	1.02
C57BR/cdJ	39.86	32.71	31.95	36.84	0.82	0.80
C57L/J	42.89	33.57	33.12	38.99	0.78	0.77
C58/J	43.61	43.52	43.02	44.15	0.99	0.98
CAST/EiJ	45.23	59.01	58.18	74.14	1.30	1.28
CBA/J	49.37	48.14	47.41	49.74	0.97	0.96
DBA/1J	38.13	30.67	30.14	37.72	0.80	0.79
DBA/2J	47.27	60.51	59.69	54.46	1.28	1.26
FVB/NJ	62.85	73.83	70.66	223.62	1.17	1.12
I/LnJ	34.55	83.82	82.27	61.33	2.42	2.38
JF1/MsJ	28.45	100.36	98.44	97.96	3.52	3.46
KK/HiJ	47.69	58.32	57.95	58.32	1.22	1.21
LEWES/EiJ	4.45	3.66	3.51	24.03	0.82	0.78
LG/J	27.89	77.63	76.74	61.34	2.78	2.75
LP/J	45.93	0.00	0.00	4.28	0.00	0.00
MOLF/EiJ	25.16	35.03	34.45	166.51	1.39	1.36
MSM/Ms	13.86	20.04	18.58	15.22	1.44	1.34
NOD/ShiLtJ	49.19	67.51	66.51	36.08	1.37	1.35
NZB/B1NJ	38.51	74.78	73.77	79.22	1.94	1.91
NZO/HlLtJ	59.11	0.01	0.01	6.69	0.00	0.00
NZW/LacJ	55.17	47.16	46.59	56.40	0.85	0.84
PWK/PhJ	44.46	95.68	94.31	71.55	2.15	2.12
RF/J	45.42	57.63	57.20	429.65	1.26	1.25
SEA/GnJ	42.12	0.00	0.00	15.28	0.00	0.00
SJL/J	40.72	35.54	35.49	24.47	0.87	0.87
SM/J	23.08	52.51	51.90	27.81	2.27	2.24
SPRET/EiJ	58.26	144.54	138.11	119.08	2.48	2.37
ST/bJ	68.09	69.69	68.52	557.64	1.02	1.00
WSB/EiJ	43.10	0.18	0.18	4.79	0.00	0.00
ZALENDE/EiJ	17.02	0.19	0.19	70.39	0.01	0.01

Reference

1. Adams, D. J., Doran, A. G., Lilue, J. & Keane, T. M. The Mouse Genomes Project: a repository of inbred laboratory mouse strain genomes. *Mamm. Genome* **26**, 403-412 (2015).
2. Takada, T. et al. The ancestor of extant Japanese fancy mice contributed to the mosaic genomes of classical inbred strains. *Genome Res.* **23**, 1329-1338 (2013).
3. Huntley, M. A. et al. Complex regulation of ADAR-mediated RNA-editing across tissues. *BMC Genomics* **17**, 61 (2016).
4. Pohl, A. A. et al. Affy exon tissues: exon levels in normal tissues in human, mouse and rat. *Bioinformatics* **25**, 2442-2443 (2009).
