# Supplementary material for: Nested retrotransposition in the East Asian mouse genome causes the classical nonagouti mutation
Source: Commun Biol. 2019 Aug 2;2:283. doi: 10.1038/s42003-019-0539-7 (PMC6677723; doi:10.1038/s42003-019-0539-7)
Supplement: Supplementary file 2 — Description of Additional Supplementary Files [file 42003_2019_539_MOESM2_ESM.docx]

**Description of Additional Supplementary Files**

**File Name**: **Supplementary Data 1**

**Description**:   **Summary of various representative exogenous and endogenous retroviruses.** Dataset showing the retroviruses information with the full name, group, data source, accession number, Repbase name, position, and length of the element and pol nucleotide used for the alignment of tree building in Figure 2e.
